# Case Report: Esophageal malignant melanoma with lung adenocarcinoma: a rare case of dual primary cancers

**DOI:** 10.3389/fonc.2025.1546806

**Published:** 2025-06-16

**Authors:** Li Yang, Fan Yang

**Affiliations:** Department of Gastroenterology, Shapingba Hospital Affiliated to Chongqing University, Chongqing, China

**Keywords:** melanoma, lung adenocarcinoma, primary malignant melanoma of the esophagus, dual primary cancers, treatment

## Abstract

Primary malignant melanoma of the esophagus (PMME) is a rare type of gastrointestinal melanoma characterized by its aggressive nature and poor prognosis, with a 5-year survival rate of less than 5%. This study reports a case of a male patient with PMME complicated by primary lung adenocarcinoma. The main symptom of the patient was progressive dysphagia. Endoscopically, a polypoid mass was observed protruding into the lumen of the lower esophagus, with melanin pigmentation on the tumor surface, part of which was smooth and part showed ulceration. Enhanced chest and abdominal CT, as well as PET-CT, were consistent with esophageal malignancy, the left lung was consistent with lung cancer, and the right was considered metastatic. CT-guided percutaneous lung biopsy and immunohistochemistry indicated left lung invasive adenocarcinoma. PMME is extremely rare, and the co-occurrence of lung adenocarcinoma as a double primary cancer is even rarer in clinical practice. The disease has a high degree of malignancy and poor prognosis, with diagnosis mainly relying on endoscopic examination, pathological histological morphology, and immunohistochemistry. Early detection and diagnosis are currently key to treating this disease.

## 1 Introduction

Primary malignant melanoma of the esophagus (PMME) is an exceedingly rare type of esophageal malignancy, accounting for less than 0.1%–0.2% of all primary malignancies of the esophagus (1, 2). It shows male predominance (3), with onset typically after 60 years of age, and frequently involves the middle to lower esophagus (4–6). Symptoms include dysphagia (7), weight loss, and chest pain (8), but preoperative diagnosis is challenging due to nonspecific presentations (9–11). PMME is highly aggressive with a poor prognosis, and the 5-year survival rate is less than 5% (12). Despite advancements in diagnostic techniques, current treatment strategies are largely based on case reports and small studies (11–14).

This report discusses a 77-year-old male with PMME and primary lung adenocarcinoma, diagnosed via gastroscopy, histopathology, and genetic testing. It explores diagnostic methods, targeted therapy, and clinical features, providing insights into PMME management.

## 2 Case description

A 77-year-old male patient with a history of hypertension and no family history of malignancy presented to the gastroenterology department on April 15, 2024, with a 2-month history of progressive dysphagia. Physical examination revealed no hyperpigmentation or macules on the skin, mucosa, sclera, or oral cavity. No palpable superficial lymphadenopathy was noted. The abdomen was soft without tenderness, rebound pain, or muscle rigidity. Laboratory tests, including complete blood count, liver and renal function panels, and tumor markers (CA15-3, CEA, AFP, CA19-9), showed no significant abnormalities.

**Figure 1:**
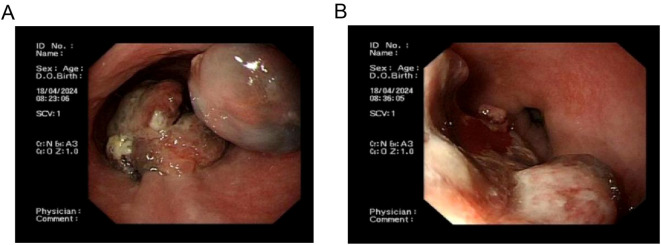
Endoscopic images of primary esophageal malignant melanoma. **(A)**, Proximal aspect of primary esophageal malignant melanoma; **(B)** Distal aspect of primary esophageal malignant melanoma.

Esophagogastroduodenoscopy revealed smooth mucosa in the upper and middle esophagus without abnormalities. At 34–36 cm from the incisors, a hemispherical lesion protruding into the lumen was observed, with well-defined borders, a smooth surface, and localized pigmentation. Additionally, at 36–39 cm from the incisors, an irregular mass with a rough, depressed surface was noted, accompanied by ulceration at the apex, covered with necrotic tissue and minimal white exudate. The lesion was friable and prone to bleeding (**Figure 1**).

**Figure 2:**
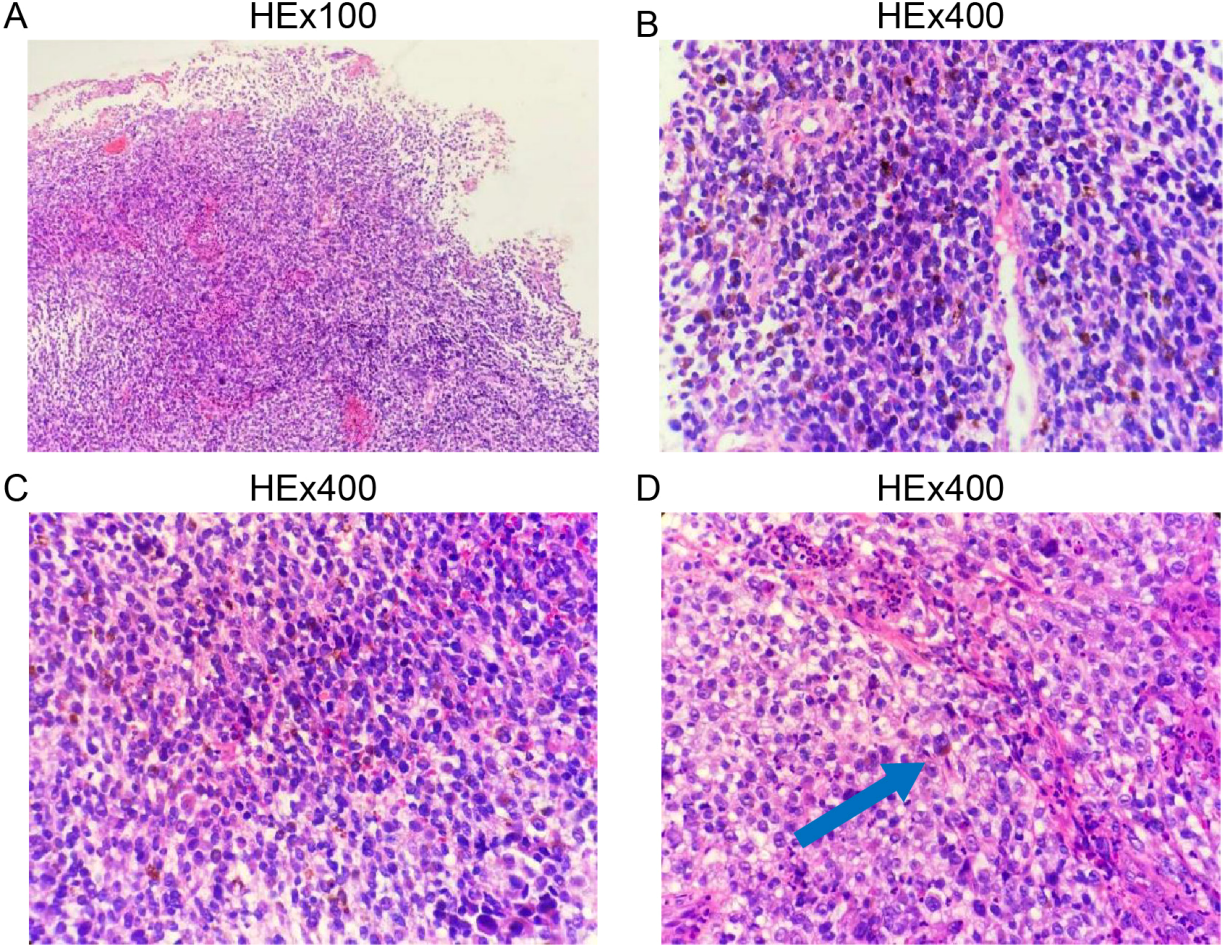
Histopathological features of primary esophageal malignant melanoma. **(A)**, Low-power magnification; **(B–D)**, High-power magnification.

Biopsy specimens from the esophageal lesions were fixed in 10% neutral formalin, routinely dehydrated, embedded in paraffin, sectioned at a thickness of 4 μm, and stained with hematoxylin-eosin (HE) and immunohistochemical markers. Microscopic examination revealed tumor cells arranged in diffuse sheets or nests. The cells were large with oval, spindle-shaped, or polygonal nuclei, prominent eosinophilic nucleoli, and frequent mitotic figures. Abundant melanin pigment was observed within and outside the cytoplasm (**Figure 2**).

**Figure 3:**
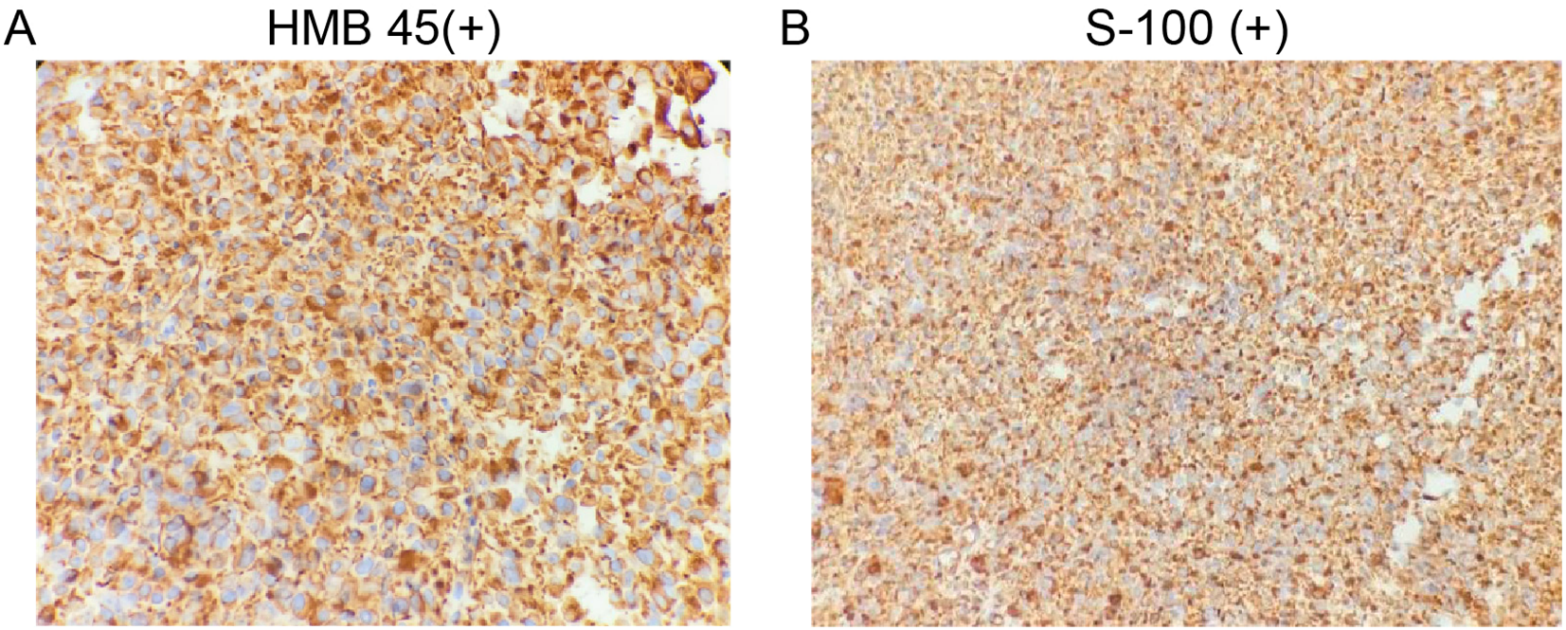
Immunohistochemical images of primary esophageal malignant melanoma. **(A)**, HMB 45(+); **(B)**, S-100(+).

Immunohistochemical staining demonstrated the following results: HMB-45 (+), SOX10 (+), Vimentin (+), Ki-67 (30–50%+), and focal positivity for P53 and S-100. Staining for CK, CK5/6, P40, P63, CgA, Syn, CD56, CEA, TTF-1, and CK7 was negative. CD34 highlighted vascular structures, and LCA was positive in lymphocytes (**Figure 3**). Based on histopathological and immunohistochemical findings, a diagnosis of PMME was established.

**Figure 4:**
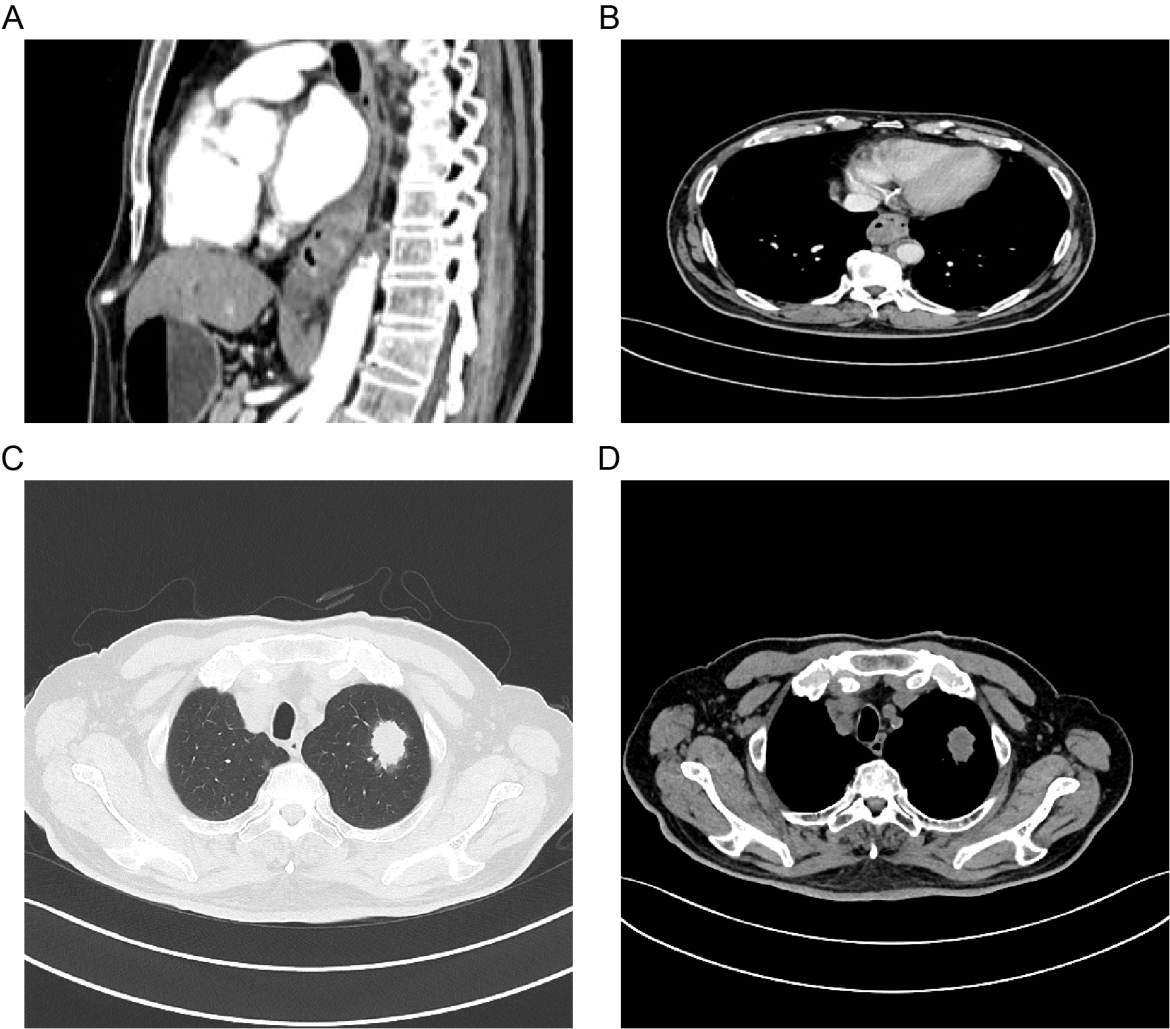
Abdominal **(A, B)** and thoracic **(C, D)** contrast-enhanced CT images.

Further molecular testing using a targeted melanoma gene panel revealed a BRAF exon 15 missense mutation (c.1799T>A, p.V600E) with a mutation allele frequency of 1.19%. Imaging studies, including contrast-enhanced CT of the chest and abdomen and PET-CT, demonstrated a malignant tumor in the distal esophagus, findings consistent with primary esophageal melanoma, as well as a lesion in the left lung suggestive of primary lung cancer and a right lung lesion indicative of metastasis (**Figure 4**). CT-guided percutaneous lung biopsy of the left upper lobe revealed invasive adenocarcinoma. Immunohistochemistry and subsequent genetic analysis identified a KRAS exon 2 mutation (c.34G>T, p.G12C) with a mutation allele frequency of 15.04%. The final diagnosis was dual primary malignancies: PMME and left lung adenocarcinoma.

The patient was initiated on targeted therapy with dabrafenib and trametinib for melanoma and treated with oral vinorelbine capsules for lung adenocarcinoma.

## 3 Discussion

PMME is a rare and aggressive form of esophageal cancer, known for its rapid spread and poor prognosis (4). It typically occurs in the middle and lower parts of the esophagus (9–11), especially the lower segment, likely due to a higher concentration of melanocytes (15, 16). Esophageal melanocytosis is seen as a major precancerous condition for PMME (15, 16), with frequent reflux possibly causing abnormal growth and cancerous changes in melanocytes.

PMME symptoms are generally vague, with initial signs like swallowing discomfort and chest pain often mistaken for poorly differentiated carcinoma (4). Advanced symptoms such as worsening swallowing difficulties, painful swallowing, weight loss, and malnutrition suggest tumor progression. Diagnosis involves ruling out metastatic esophageal melanoma, poorly differentiated squamous cell carcinoma, and sarcomatoid carcinoma.

PMME predominantly depends on endoscopic evaluation, histopathological analysis, and immunohistochemical techniques. Endoscopically, PMME typically manifests as an intraluminal polyp, frequently exhibiting pigmentation (12). Pathological diagnosis involves identifying melanin granules and using melanoma markers such as S-100, SOX-10, and HMB-45 to improve diagnostic accuracy (17–19).

Esophagectomy is the main treatment for PMME, as some studies suggest it may prolong survival (3). However, because PMME is often diagnosed late or with metastases, surgery alone has limited effectiveness (20, 21). Adjuvant therapies like radiotherapy, chemotherapy, and immunotherapy can enhance local control, but their effectiveness varies by individual. Recently, the use of genetic testing and targeted therapies in PMME has grown. For patients with BRAF V600 mutations, the American Joint Committee advises treatment with BRAF inhibitors (dabrafenib, vemurafenib, encorafenib) and MEK inhibitors (trametinib, cobimetinib, binimetinib) (22).

In short, PMME is an aggressive esophageal tumor with a poor prognosis. Diagnosis involves endoscopy, pathology, and molecular tests. Surgery is the primary treatment, but combined therapies like chemoradiotherapy, immunotherapy, and targeted therapy can enhance survival. Early detection and precise diagnosis are crucial for better survival rates.

## Data Availability

The original contributions presented in the study are included in the article/supplementary material. Further inquiries can be directed to the corresponding author.
